# The role of capillary transit time heterogeneity in myocardial oxygenation and ischemic heart disease

**DOI:** 10.1007/s00395-014-0409-x

**Published:** 2014-04-18

**Authors:** Leif Østergaard, Steen Buus Kristiansen, Hugo Angleys, Jørgen Frøkiær, J. Michael Hasenkam, Sune Nørhøj Jespersen, Hans Erik Bøtker

**Affiliations:** 1 Department of Neuroradiology, Aarhus University Hospital, Building 10G, Nørrebrogade 44, 8000 Aarhus C, Denmark; 2 Center of Functionally Integrative Neuroscience and MINDLab, Aarhus University, Building 10G, Nørrebrogade 44, 8000 Aarhus C, Denmark; 3 Department of Cardiology, Aarhus University Hospital, Brendstrupgaardsvej 100, 8200 Aarhus N, Denmark; 4 Department of Nuclear Medicine and PET-Center, Aarhus University Hospital, Brendstrupgaardsvej 100, 8200 Aarhus N, Denmark; 5 Department of Cardiothoracic and Vascular Surgery, Aarhus University Hospital, Brendstrupgaardsvej 100, 8200 Aarhus N, Denmark; 6 Department of Physics and Astronomy, Aarhus University, Ny Munkegade 120, 8000 Aarhus C, Denmark

**Keywords:** Microvascular dysfunction (MVD), Ischemic heart disease (IHD), Microcirculation, Oxygen transport, Myocardial blood flow (MBF), Capillary transit time heterogeneity (CTH), Reperfusion injury, Myocardial capillaries, Glycocalyx, Connexins, Pericyte

## Abstract

Ischemic heart disease (IHD) is characterized by an imbalance between oxygen supply and demand, most frequently caused by coronary artery disease (CAD) that reduces myocardial perfusion. In some patients, IHD is ascribed to microvascular dysfunction (MVD): microcirculatory disturbances that reduce myocardial perfusion at the level of myocardial pre-arterioles and arterioles. In a minority of cases, chest pain and reductions in myocardial flow reserve may even occur in patients without any other demonstrable systemic or cardiac disease. In this topical review, we address whether these findings might be caused by impaired myocardial oxygen extraction, caused by capillary flow disturbances further downstream. Myocardial blood flow (MBF) increases approximately linearly with oxygen utilization, but efficient oxygen extraction at high MBF values is known to depend on the parallel reduction of capillary transit time heterogeneity (CTH). Consequently, changes in capillary wall morphology or blood viscosity may impair myocardial oxygen extraction by preventing capillary flow homogenization. Indeed, a recent re-analysis of oxygen transport in tissue shows that elevated CTH can reduce tissue oxygenation by causing a functional shunt of oxygenated blood through the tissue. We review the combined effects of MBF, CTH, and tissue oxygen tension on myocardial oxygen supply. We show that as CTH increases, normal vasodilator responses must be attenuated in order to reduce the degree of functional shunting and improve blood-tissue oxygen concentration gradients to allow sufficient myocardial oxygenation. Theoretically, CTH can reach levels such that increased metabolic demands cannot be met, resulting in tissue hypoxia and angina in the absence of flow-limiting CAD or MVD. We discuss these predictions in the context of MVD, myocardial infarction, and reperfusion injury.

## Introduction

**Fig. 1:**
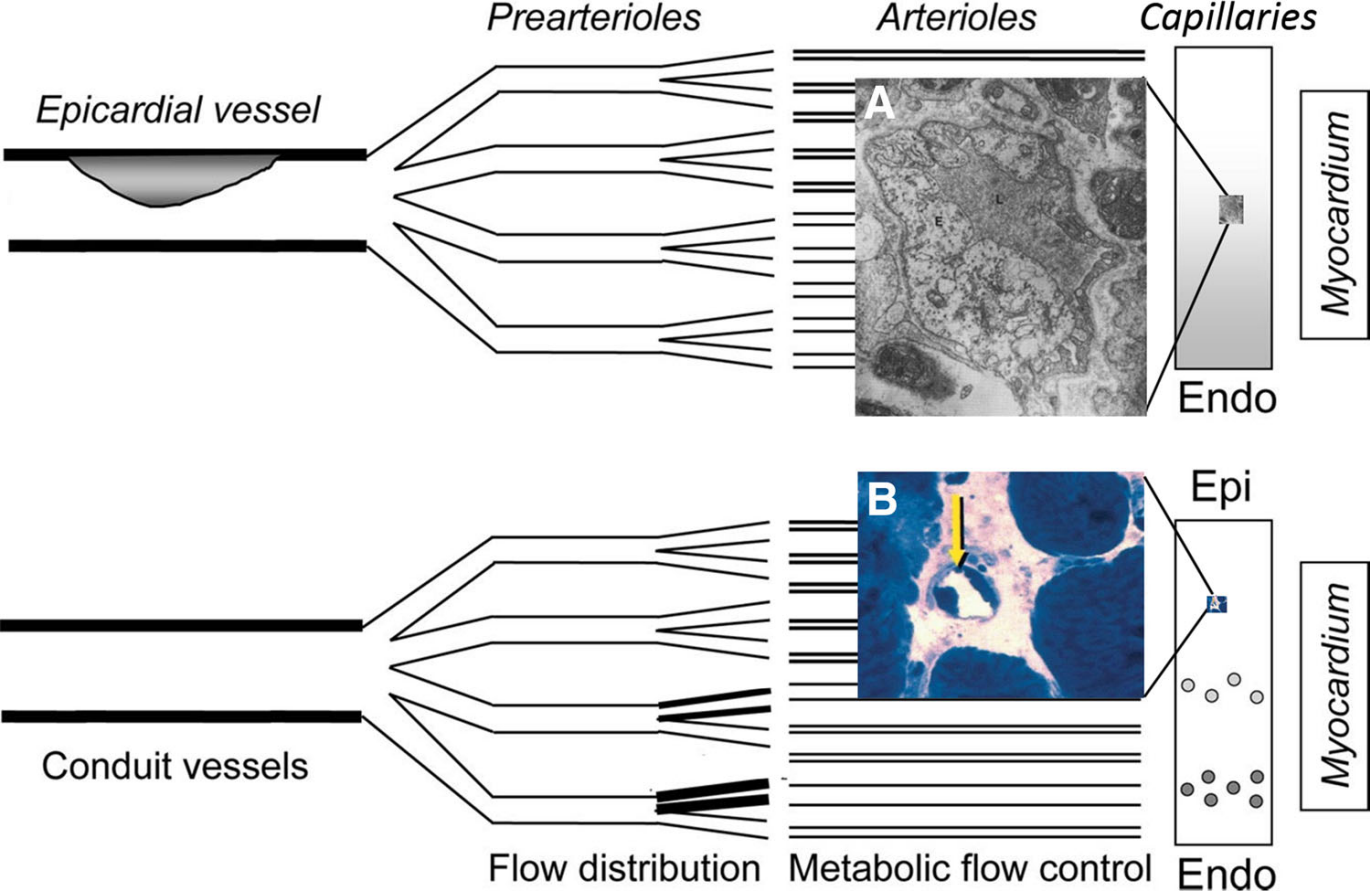
Vascular changes in ischemic heart disease and its risk factors. The figure illustrates the three levels at which the vascular system is affected in cardiovascular risk factors: at the level of coronary arteries, (pre-) arterioles, and capillaries. Modified from a figure by Lanza et al. [64]—see also Table 1. *Insets* show **A** narrowing of the capillary lumen due to swelling and degenerative changes in the endothelial cells from a patient with exertional angina but patent coronary arteries [74] and **B** glycosphingolipid inclusions in the capillary endothelium in Fabry’s disease [31]. Figures are reproduced with permission from the publishers

Ischemic heart disease (IHD) can be viewed as an imbalance between oxygen supply and demand [41]. The condition is most frequently caused by reductions in the blood supply to the heart muscle due to coronary artery disease (CAD). Accordingly, patients who suffer from episodes of chest pain typically display obstructive atherosclerotic lesions in their epicardial arteries and reductions in coronary flow reserve (CFR)—the myocardial blood flow (MBF) response to physiological (reactive hyperemia) and pharmacological vasodilators [43]. In some patients, chest pain develops in the absence of significant CAD. It is now recognized that disturbances in the myocardial microcirculation can be the source of IHD in some patients; a condition referred to as microvascular dysfunction (MVD) [20]. These microcirculatory disturbances are caused by morphological changes in the walls of myocardial pre-arterioles and arterioles, myocardial abnormalities (e.g., hypertrophy, deposits, or infiltrating disorders) that compress myocardial microvessels, rheological disorders that increase blood viscosity [99] or, in the iatrogenic form of MVD, by microembolizations after revascularization procedures [44]—see Fig. 1. MVD is characterized by reduced responses to vasodilators and exaggerated responses to vasoconstrictors, and often occur in relation to cardiovascular risk factors such as smoking, hypertension, hyperlipidemia, diabetes [64], and other insulin-resistant states [12]. Importantly, MVD can coexist with CAD and contribute to high morbidity after appropriate management of large vessel disease [64].

In some patients, or in some phases of the disease, MVD appears to be associated with disturbances in myocardial oxygen extraction, rather than reductions in MBF per se. In patients with diabetes and hypertension, chest pain and reductions in CFR can develop prior to the onset of their microvascular complications. Indeed, some MVD patients show no demonstrable systemic or cardiac disease, a disease entity previously referred to as cardiac syndrome X although it is important to recognize that only 40 % of this heterogeneous patient group have signs of MVD [13]. Taken together, these MVD subtypes challenge the notion of IHD as a flow-limiting condition only. In fact, studies have reported elevated MBF, rather than hypoperfusion, throughout the myocardium in patients with diabetes [72], and in some regions of the myocardium in patients with cardiac syndrome X [70]. But how can oxygen extraction in the myocardium be limited when MBF is unhindered?

### The effect of capillary blood flow patterns on the extraction of oxygen in the myocardium

**Fig. 2:**
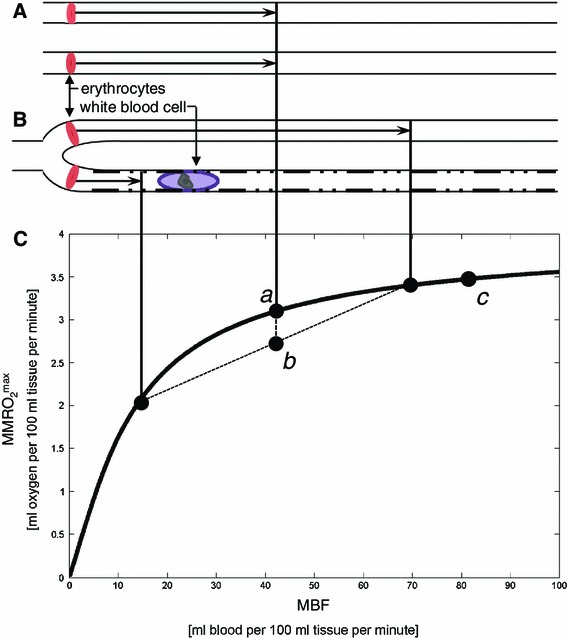
The classical flow-diffusion equation for oxygen. The classical flow-diffusion equation curve (**C**) shows the maximum amount of oxygen that can diffuse from capillaries to tissue for a given perfusion rate, under the assumption that all erythrocytes pass through the tissue capillaries at identical velocities, as indicated in **A**. This assumption is rarely considered, but the heterogeneous distribution of capillary flows shown in **B** shows why this assumption is important: because of the shape of the curve in **C**, it is an inherent property of the classical flow-diffusion that it overestimates tissue oxygenation if capillary flows are heterogeneously distributed [89]. This is seen by using the curve, which is accurate for individual capillaries, to determine the net tissue oxygen availability resulting from the individual flows in case **B**. The resulting net tissue oxygen availability is the weighted average of the oxygen availabilities for the two flows, labeled *b* in the plot. Note that the resulting tissue oxygen availability will always be less than that of the homogenous case, labeled *a*. In fact, capillary flows are heterogeneous in normal, resting tissue, but homogenize during hyperemia. Label *c* shows a condition of higher flow, with homogenous capillary flow. Note how homogenization + hyperemia (*b* *→* *c*) provides a larger increase in tissue oxygenation than hyperemia with homogenous capillary flow (*a* *→* *c*) as assumed by the classical flow-diffusion equation. The hindered capillary passage indicated in the figure is the sum of preexisting age- or risk-factor-related changes, and ischemia-related changes such as altered blood–cell interactions with the endothelial surface properties (cell adhesion, loss of glycocalyx, and so forth), and/or external edema pressure. Modified from [79]

Rose and colleagues first demonstrated that the efficient extraction of diffusible substances in the myocardium at high MBF can be explained by concomitant reductions in capillary transit time heterogeneity (CTH), as derived from indicator dilution studies [19, 89]. Figure 2 illustrates this phenomenon for oxygen: The microcirculation of the normal myocardium is heterogeneously perfused during rest [84], and due to the extraction properties of single capillaries, oxygen extraction efficacy can therefore be improved by homogenizing capillary flow patterns as MBF increases. However, capillary flow patterns depend critically on the topology and morphology of the capillary bed [85, 86], and even minor disturbances in capillary wall function or blood viscosity would therefore be expected to impair myocardial oxygen extraction, particularly during episodes of increased MBF. Below, we review evidence of altered capillary morphology and blood viscosity that could cause CTH to increase in MVD. Then, we use a model oxygen transport in tissue that takes the effects of CTH into account [52] to predict which clinical findings would be expected to result from a gradual increase of CTH. The oxygen transport model is described in detail in [52], and its application to cerebral ischemia in [79].

### Regulation of capillary blood flow

The heterogeneity of capillary transit times during rest and their homogenization towards high flow rates may be a passive effect of capillary bed topology and morphology [85, 86]. The capillary wall, however, also contains contractile pericytes [90] which form the functional unit of the capillariomotor system; a mechanism that seemingly ensures the redistribution of erythrocytes along capillary paths according to the regional oxygen needs of the tissue [61]. Indeed, retinal pericytes constrict in response to high oxygen tension but relax in response to lactate and low pH [118], possibly providing a mechanism by which pericytes can redistribute capillary flows according to local cellular metabolic needs in a continuous manner during rest [118]. Pericytes are embedded in the capillary basement membrane and have been studied extensively in muscle tissue [15]. Organ perfusion studies suggest that pericytes from skeletal muscle, but not myocardial pericytes, constrict upon exposure to angiotensin, norepinephrine, and vasopressin [103]. Unlike pericytes from skeletal muscle tissue, however, myocardial pericytes are often found in close relation to myocardial nerve terminals [102]. The way in during which central innervation and local vasoactive signals regulate pericyte tone in vivo, however, remains poorly understood. Studies of retinal capillaries suggest that pericytes react to intrinsic signaling in much the same way as smooth muscle cells: Pericyte constrictions have hence been observed in response to mechanical stretch and exposure to angiotensin II (via AT_1_ receptors) [55] and endothelin-1 (via ET_A_ receptors) [91], by a Ca^++^ dependent mechanism [25]. Meanwhile, pericytes relax in response to adenosine [68], ATP [56], and nitric oxide (NO) [37, 38], as well as to cholinergic [117] and adrenergic (via β_2_ receptors) [25] stimulation. Importantly, recent studies show that cerebral pericytes control blood flow, while ischemia and oxidative stress cause irreversible constrictions of cerebral pericytes [39, 120].

The luminal surface of the capillary endothelium is covered by a 0.5-µm-thick carbohydrate-rich matrix, the glycocalyx [111], which affects the passage of blood cells through the capillary bed [93]. Electrostatic interactions between erythrocytes and glycocalyx [112] and slow passage of plasma in relation to this endothelial surface layer [60] reduce capillary hematocrit to 20–50 % of that found in the systemic circulation, and disruption of the glycocalyx therefore causes capillary hematocrit to approach that of the systemic circulation [18, 23]. The glycocalyx constitutes a fluid barrier in the vascular system [105, 106], and glycocalyx degradation is hence associated with myocardial edema [105], and possibly capillary compression. The glycocalyx is degraded by exposure to direct oxidative stress and oxidized lipoproteins [18, 21, 110], to acute hyperglycemia [77], and ischemia [21, 51]. Disruption of the glycocalyx alters the normal blood flow responses and hence the normal relation between blood flow and metabolism in the myocardium [108, 109]. While glycocalyx disruption and disturbed capillary flow patterns often occur in parallel, it is unclear whether these changes are causally related [22].

Endothelial cells throughout the vascular system are electrically and metabolically coupled to each other, and to nearby smooth muscle cells, via gap junctions composed of so-called connexins [50]. The extent to which pericytes are also involved in this efficient vascular signaling is uncertain, but this rapid, bidirectional signaling pathway via gap junctions seemingly ensures efficient coordination of vessel function across the microvascular bed [27, 94, 95].

### Changes in capillary morphology and blood viscosity in MVD

At the level of myocardial capillaries, conditions that predispose to MVD are associated with changes in capillary wall morphology, including basement membrane thickening, endothelial swelling, or compression by the surrounding tissue—see Fig. 1 and Table 1. These changes are observed in capillaries that appear to have been perfused and would hence be expected to disturb—rather than to block—the capillary distribution of erythrocytes. Similarly, increased blood viscosity and abnormal blood cell adhesion to the capillary endothelium would be expected to disturb the regulation of capillary flow patterns, rather than to block myocardial perfusion. As illustrated by Fig. 2, the dimensions of white blood cell (WBC) and erythrocyte exceed the average capillary diameter. Experimental studies have shown that capillary flow patterns are sensitive to the size, viscosity, number, and endothelial adhesion of blood cells, and undergo profound changes during infections [69] and as part of the low-grade vascular inflammation that accompany many cardiovascular risk factors [69, 110].

**Table 1:** Changes in capillary morphology in MVD risk factors

Risk factor	Changes in capillary morphology	Reference(s)
Dilated and hypertrophic cardiomyopathy (human)	Irregular capillary diameters. Frequent narrowing of the capillary lumen due to thickened endothelial cells ~20 % of capillaries affected	[74, 100]
Diabetes (human)	Thickening of basement membrane	[34]
SVD without CAD (human)	Reduced capillary diameters. Frequent narrowing of the capillary lumen due to thickened endothelial cells. ~50 % of capillaries affected	[74]
Chagas disease (human)	Basement membrane thickening	[33]
Fabry’s disease (animal model)	Glycosphingolipid inclusions in the capillary endothelium	[31]
Smoking	Nicotine up-regulates the expression of adhesion molecules in the capillary endothelium and increases leukocyte rolling	[2, 122]

### Relation between MBF, CTH, oxygen tension, and the extraction of oxygen

**Fig. 3:**
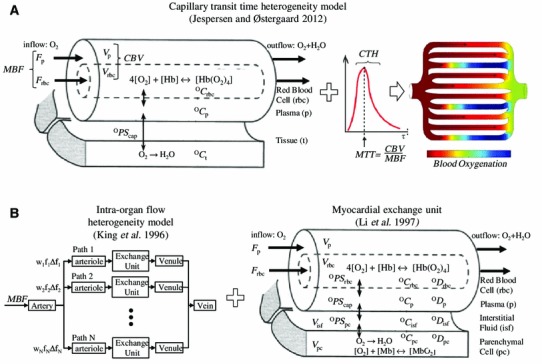
Models of myocardial oxygenation and myocardial oxygen utilization. Figure 3A shows the recent model of the effects of capillary transit time heterogeneity (CTH) and myocardial blood flow (MBF) on myocardial oxygen availability. The model assumes the well-established properties of single capillaries (Fig. 2), but instead of assuming that all tissue capillaries have equal flows (as the traditional flow-diffusion equation), it assumes a realistic distribution of capillary transit times (*red curve*) to infer the net oxygen availability. The model takes into account (*left panel*) the binding of oxygen to hemoglobin (Hb) by applying the Hill equation, but not the effects of local pH changes (the Bohr effect) when determining the plasma concentration of oxygen (^O^C_p_) based on that of blood (^O^C_rbc_). The model assumes either fixed capillary blood volume (CBV) or a blood volume that gradually increased with MBF (recruitment). The model assumes a single transfer constant for oxygen between blood and tissue (dubbed ^O^PS_cap_ in the figure to facilitate comparison with the oxygen transport model in Fig. 3B). We assume steady-state conditions (i.e., oxygen is metabolized at the rate it is extracted from capillaries), and that the concentration of oxygen in tissue (^O^C_t_) is constant. The model thereby attempts to predict the maximal myocardial oxygen extraction fraction (OEF^max^) and metabolic rate of oxygen (MMRO_2_^max^) that the vasculature can support at certain levels of MBF and CTH, rather than predicting blood or tissue oxygen levels by which measured, labeled oxygen levels can be fitted to infer tissue metabolism (see Fig. 3B). The predictions of OEF^max^ and MMRO_2_^max^ are shown in Figs. 5 and 6 for various combinations of MBF, CTH, and tissue oxygen tension, while the various combinations of these parameters that provide sufficient oxygenation to meet the metabolic needs of the resting heart are visualized in Fig. 7. Figure 3B illustrates how models of the regional flow distribution (*left*) have been combined with models of oxygen exchange (*right*) to model the transport and/or metabolism of intravascular and diffusible tracers in the heart; in this case oxygen. The objective of these models is to permit the determination of physiological variables such as myocardial blood flow (for example, myocardial perfusion imaging using MRI-based residue detection of an intravascular tracer [63]) or oxygen metabolism (for example, by ^15^O_2_ injection, and subsequent measurements of tracer distribution by either venous outflow measurements or PET [24, 92]) by fitting experimental data to the appropriate model. This model can incorporate regional myocardial flow heterogeneity by varying the weights (*w*_i_) and relative flows (*f*_i_∆*f*_i_) through the parallel paths (*left*), just as all vascular tracer transport (through artery, arteriole, venule, and vein) contains a relative dispersion (RD) term, which is generally kept constant [58]. Note that, even if this vascular transport model was combined with the oxygen transport model on the right and applied capillary flow heterogeneity [78], there is no straightforward way of setting or summarizing the many model variables in a way that would permit an intuitive understanding of the effects of varying CTH. A general, conceptual drawback of this model is the dependency of the model ‘constants’ PS on blood flow in biological systems—see text. Figures modified from Jespersen and Østergaard [52], King et al. [58], and Li et al. [65] with permissions from the publishers. Abbreviations: ^*O*^ *PS* permeability surface area product for oxygen—subscripts denote the corresponding biological barrier as indicated in the figure, ^*O*^ *C* concentration of oxygen (subscripts denote the corresponding tissue compartment as indicated in the figure), ^*O*^ *D* radial diffusivity of oxygen (subscripts denote the corresponding tissue compartment as indicated in the figure), *Mb* myoglobin, *Hb* hemoglobin, *Hb*(*O*_*2*_)_*4*_ oxyhemoglobin, *F*_p_ plasma flow, *F*_*rbc*_ flow of red blood cells, *V*_*p*_ plasma volume, *V*_*rbc*_ capillary red blood cell volume (hematocrit), *PET* positron emission tomography, *MRI* magnetic resonance imaging

**Fig. 4:**
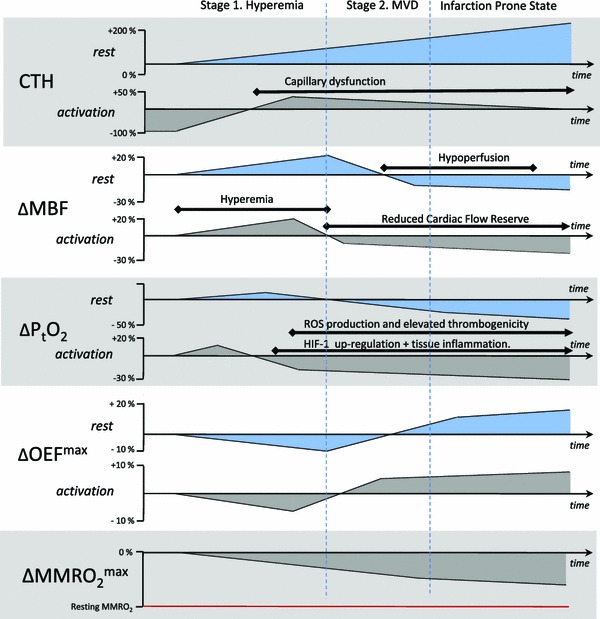
Changes in MBF and tissue oxygen tension that must accompany increasing levels of capillary dysfunction in order to maintain tissue oxygen availability. The figure displays the adaptations of MBF and P_t_O_2_ that are necessary in order to maintain tissue oxygen availability as CTH levels gradually increase over time. The gradual reduction in MFR is indicated in the lower panel. In the infarction-prone state, minor reductions in MBF or increases in CTH are predicted to result in symptoms as MMRO_2_^max^ approaches the actual, metabolic needs of the tissue. Abbreviations: *MBF* myocardial blood flow, *P*_*t*_ *O*_*2*_ tissue oxygen tension, *CTH* capillary transit time heterogeneity, *MMRO*_*2*_^*max*^ maximum myocardial metabolic rate of oxygen

The relation between blood flow (measured in mL blood per 100 mL tissue per minute) through tissue, and its access to oxygen, is traditionally derived from the classical flow-diffusion equation [88] depicted by the curve in Fig. 2. While this equation is only accurate for single capillaries, or in tissue with homogenously perfused capillaries, the complexity of describing systems with multiple capillaries and transit times has thus far prevented the study of tissue oxygenation changes in response to increasing CTH. Figure 3A describes our first attempt to develop a model of tissue oxygenation that incorporates the effects CTH by assuming a distribution of capillary transit times [52]. We used the accepted gamma variate function, according to which CTH is introduced as a single parameter, namely the standard deviation of capillary transit times. Figure 4 illustrates how MBF and myocardial tissue oxygen tension (P_t_O_2_) must be adapted in order to maintain myocardial oxygenation in response to a gradual increase in CTH (top row). Below, we describe the tissue oxygenation model [52] and the origin of these predictions in greater detail, and then discuss the model and its properties in relation to existing biophysical models of myocardial oxygen transport.

**Fig. 5:**
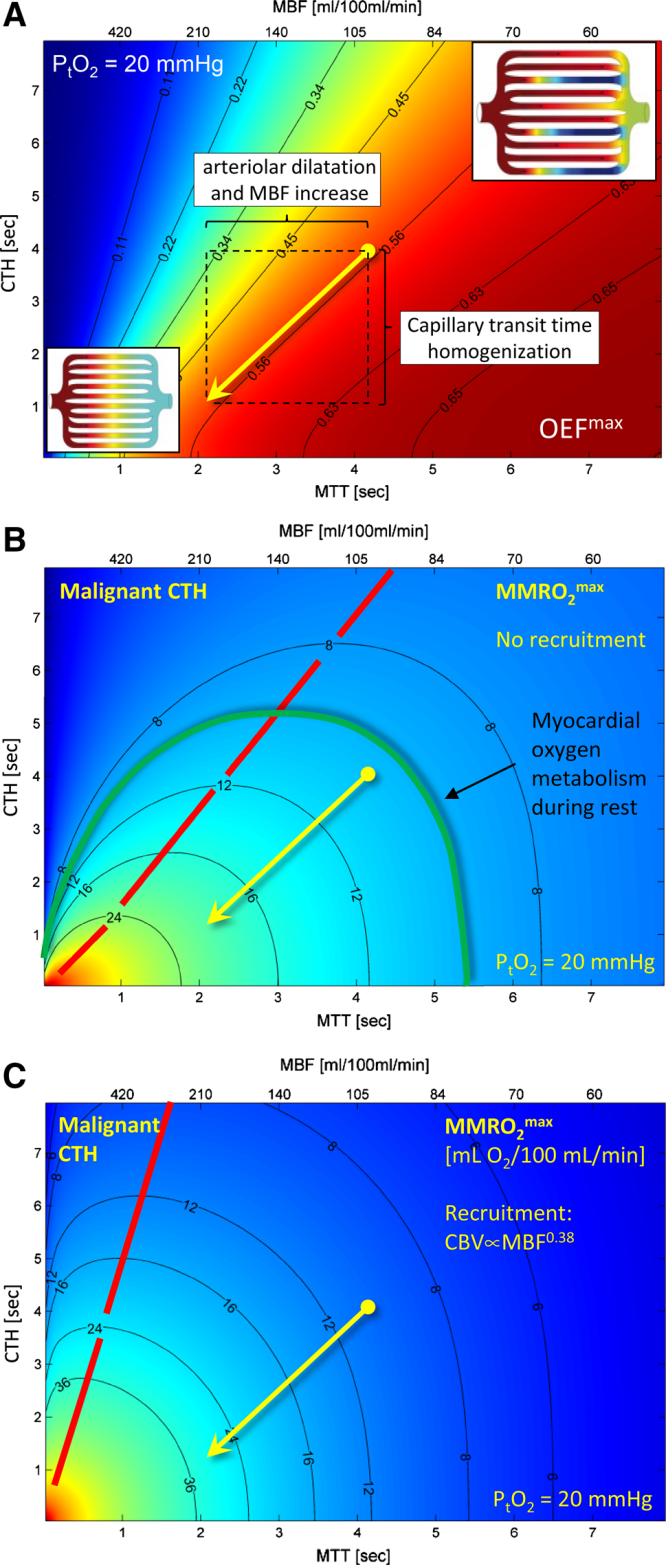
Effects of mean transit time, capillary transit time heterogeneity, tissue oxygen tension, and capillary recruitment on oxygen extraction. **A** shows a contour plot of OEF^max^ as a function of MTT and CTH. The oblique arrow indicates the change in CTH which must occur during a doubling of MBF in order to maintain oxygen extraction fraction. The corresponding tissue oxygen availability, MMRO_2_^max^ is shown in units of mL/100 mL/min for constant CBV in **B**, and for capillary recruitment in **C**. In hemodynamic states above the *yellow line* in **B** and **C**, increases in MBF will lead to reductions of tissue oxygen availability. Abbreviations: *MTT* Mean transit time, *CTH* capillary transit time heterogeneity, *P*_*t*_ *O*_*2*_ tissue oxygen tension, *OEF* ^*max*^ maximum oxygen extraction fraction, *MBF* myocardial blood flow, *MMRO*_*2*_^*max*^ maximum myocardial metabolic rate of oxygen

Figure 5 illustrates the properties of the extended flow-diffusion model, based on the hemodynamics and the metabolic demands of the myocardium [52]. For convenience, we summarized myocardial hemodynamics both in terms of MBF (secondary *x*-axis) and the mean transit time (MTT) for blood as it passes through myocardial capillaries (primary *x*-axis). According to the central volume theorem [98] MTT equals the myocardial capillary blood volume (CBV), which we set to 7 % [8], divided by MBF. The contour plot in Fig. 5A shows the maximum oxygen extraction fraction (OEF^max^) that can be achieved for any combination of CTH and MTT (or MBF) for a fixed tissue oxygen tension (P_t_O_2_) of 20 mmHg. The value of OEF^max^ that corresponds to a given location in the (MBF, CTH) plane is most easily derived from the OEF^max^ values indicated on the two nearest iso-contours.

The diagonal arrow in Fig. 5A indicates a doubling of MBF from 100 mL/100 mL/min to 200 mL/100 mL/min at a constant OEF^max^ of 55 %, and constant P_t_O_2_. In the human left ventricle, OEF values range 65–75 % during rest [71], while 4–5 fold increases in myocardial oxygen consumption during heavy exercise are met by increases in MBF, and to a small extent in OEF [26, 42, 46]. In the right ventricle, animal studies suggest that OEF is <50 % during rest, while increases in OEF account for the majority of the increase in oxygen availability during heavy exercise [40]. Accordingly, the arrow in Fig. 5A illustrates how left ventricle can maintain constant OEF^max^ during several-fold increases in MBF without reductions in myocardial oxygen tension [28]. Similarly, a concomitant increase in OEF can be achieved at fixed P_t_O_2_ by larger reductions in CTH during the MBF increase. The horizontal component of the arrow (indicated as a horizontal dashed line in Fig. 5A) indicates the OEF^max^ change that would result from a vasodilation alone, assuming that the CTH and P_t_O_2_ remained fixed during the increase in MBF. Note that if CTH cannot be reduced, OEF^max^ decreases as MBF increases, owing to the poor extraction of oxygen from capillaries with very short transit times. This model property is consistent with the original observations by Rose and colleagues, namely that CTH must be reduced at high MBF in order to explain the efficient extraction of solutes by the myocardium during vasodilation [19, 89].

### Net oxygen extraction capacity in the myocardium, MMRO_2_^max^: capillary recruitment

Figure 5B and C shows contour plots of the maximum myocardial metabolic rate of oxygen (MMRO_2_^max^, measured in mL O_2_/100 mL tissue/min) that can be supported for any combination of MTT and CTH, again for a fixed tissue oxygen tension of 20 mmHg. These figures are derived from 5A by multiplying its OEF^max^ values by a typical arterial oxygen concentration (19 mL/100 mL), and MBF. In Fig. 5B, we assumed that all myocardial capillaries are perfused, i.e., absence of capillary recruitment (opening of previously un-perfused capillaries) at high flow rates. In Fig. 5C, we assumed that capillaries were recruited as a function of increasing MBF. Specifically, CBV was set to increase as the 0.38th power of MBF, implying a 30 % increase in CBV as MBF increases from 100 to 200 mL/100 mL/min, and a 52 % CBV increase when MBF triples. Note that, with this degree of capillary recruitment, a doubling of MBF at constant CTH (horizontal arrow in Fig. 5C) improves tissue oxygen availability as much as doubling MBF with a parallel CTH reduction without recruitment (oblique arrow in Fig. 5B). This effect is discussed further below.

The extent to which hyperemia is accompanied by capillary recruitment in the heart remains uncertain. Both a reduction of CTH, and capillary recruitment, increase the effective capillary surface area for solute extraction [52], and classical indicator dilution studies have therefore not been able to prove or disprove the existence of recruitable capillaries—see discussion in Reference [29]. The oxygen extraction fraction (OEF) in the myocardium is constant, or even increases, as MBF increases [28]. According to Fig. 5A, this observation implies that hyperemia is accompanied by either a CTH reduction along the oblique lines in Fig. 5A–C, or by constant CTH and MTT. The maintenance of constant MTT would imply that capillaries are recruited such that CBV increases in proportion to both MBF and the net myocardial oxygen utilization. Indicator dilution studies suggest, however, that relative CBV increases are only half as big as the corresponding increases in myocardial oxygen consumption [29]. Therefore, it seems that a reduction of CTH, but not capillary recruitment, is necessary to explain the observed coupling between MBF and myocardial oxygen metabolism. Below, we therefore limit our description of the extended flow-diffusion model to include its features in the absence of capillary recruitment.

### Stage 1 Ensuring myocardial oxygenation as CTH increases: adaptations to small increases in CTH

Using CTH as a parameter that summarizes the effects of disturbed capillary flow patterns, we can now analyze the effects of ‘capillary disease’ on myocardial oxygenation, isolated from the effects that MVD may have on arterial and arteriolar patency, and thereby MBF and MTT. We refer to increases in CTH, and the accompanying inability to reduce CTH (homogenize capillary flow patterns), as capillary dysfunction below. Figure 4 provides an overview of the dynamics changes in MBF, P_t_O_2_, OEF^max^, and MMRO_2_^max^ that follow from a gradual increase in CTH.

For small increases in CTH, the parallel reduction in OEF^max^ can be compensated by increases in MBF to meet the metabolic needs of the myocardium. The prediction that early changes in capillary morphology give rise to less efficient oxygen extraction is consistent with findings of reduced oxygen extraction fractions (OEF) in the myocardium of rats with streptozotocin-induced diabetes [82], in diabetic patients [4], and in patients with microvascular angina [13], as well as with findings of increased regional MBF during rest in patients with diabetes and syndrome X [14, 70, 72].

### Stage 2 Adapting MBF, and blood flow responses, to larger increases in CTH

Perhaps the most critical property of capillary dysfunction is that as MTT decreases (MBF increases), vasodilation may fail as a means of increasing the availability of oxygen in the myocardium at a given tissue oxygen tension. This phenomenon is associated with combinations of MTT (MBF) and CTH that lie left of the red line in Fig. 5B, and we refer to levels of CTH that are so high relative to MTT, as malignant CTH [52]. The hemodynamic conditions under which this phenomenon arises depend on the distribution of transit time in the tissue, but physiologically, it should be understood as a condition in which increasing amounts of blood oxygen becomes inaccessible to the tissue due to the short transit time during which blood is available for blood-tissue oxygen exchange [47]. The resulting reduction in OEF^max^ eventually outweighs the increase in passing, oxygenated blood. Continued oxidative metabolism in the myocardium will reduce oxygen tension in the tissue under such conditions, making blood-tissue concentration gradients higher, and oxygen extraction more efficient. To avoid the excessive, functional shunting of blood, however, normal vasodilatory responses to tissue hypoxia must be attenuated: otherwise, incremental vasodilation would tend to reduce oxygen availability further, lower oxygen tension yet more, and so on, in a vicious cycle.

**Fig. 6:**
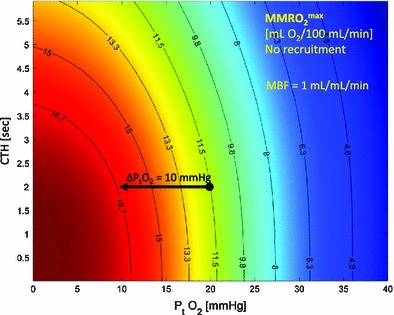
Myocardial oxygen availability without reactive hyperemia, at normal MBF. MMRO_2_^max^ is plotted as a function of tissue oxygen tension and CTH for constant MBF (100 mL/100 mL/min). Note that a reduction in tissue oxygen tension of 10 mmHg correspond to a 50 % increase in MMRO_2_^max^. Therefore, if MBF remains suppressed, the increased blood-tissue oxygen concentration gradients that accompany the increased oxygen metabolism during increased workload can facilitate the extraction of oxygen in amounts that are sufficient to support additional energy needs of the tissue. Abbreviations: *MMRO*_*2*_^*max*^ maximum myocardial metabolic rate of oxygen, *CTH* capillary transit time heterogeneity, *MBF* myocardial blood flow

Provided that MBF responses can be suppressed, Fig. 6 illustrates how low tissue oxygen can facilitate the extraction of oxygen: the net oxygen extraction (MMRO_2_^max^) is plotted as a function of tissue oxygen tension and CTH for a constant MBF of 100 mL/100 mL/min, which is typical of myocardium during rest. Note that a reduction in tissue oxygen tension of 10 mmHg corresponds to a 40 % increase in MMRO_2_^max^. Therefore, provided MBF remains suppressed, the increased blood-tissue oxygen concentration gradients that accompany the additional oxygen metabolism during an increased workload can by themselves facilitate the extraction of oxygen in amounts that are sufficient to support substantial increases in myocardial workload.

Paradoxically, the progressive suppression of MBF responses, reduced MFR, and gradual reductions in tissue oxygen tension, are predicted to be necessary means of preserving tissue oxygen availability when ageing and cardiovascular risk factors cause CTH to increase over time (cf. Table 1). Under these conditions, tissue oxygen tension is predicted to drop further during episodes of increased metabolic demands, and therefore ultimately cause angina. As indicated in Fig. 4, elevated CTH as a result of capillary flow disturbances thereby present with the characteristic features of MVD. This prediction is consistent with the findings of reduced MFR in some cases of MVD and cardiac syndrome X [11, 64] and with the finding that reductions in MFR are more frequent in patients with small vessels disease [13], in whom changes in capillary morphology are more severe [74]—see Table 1. One might expect that reduced tissue oxygen tension would elicit angiogenesis and hence a ‘physiological’ recruitment of new vessels to improve tissue oxygenation. It should be kept in mind, however, that existing capillaries are the main source of the elevated CTH. The formation of new, low-resistance capillary paths might in fact be suspected to increase arteriolo-venular shunting rather than to improve tissue oxygenation [80].

In Fig. 4, we illustrated the hypothesized improvement in OEF^max^ that results from suppression of MBF. We indicated a curve that eventually exceeds the OEF of healthy, left ventricle myocardium. The extent to which a relatively hypoxic myocardium can facilitate OEF values that are above those of normal myocardium remains poorly understood. Messer et al. [71] found reduced OEF (*N* = 20, 66 % compared to 70 % in controls) in patients with coronary artery disease, but these patients were able to progressively increase OEF after 3 (73 %) and 7 (75 %) min of exercise, without any increase in lactate levels. These findings are consistent with the notion that relative myocardial hypoxia may increase OEF to supranormal values. Similar findings were reported by Holmberg et al. [46] who measured smaller MBF increase during exercise in patients with coronary insufficiency than in controls (222 vs. 271 %), but correspondingly larger increases in OEF in the patients (76 vs. 66 % in normal controls).

### Stage 3 Critical increases in CTH: angina and proneness for acute cardiac syndrome

**Fig. 7:**
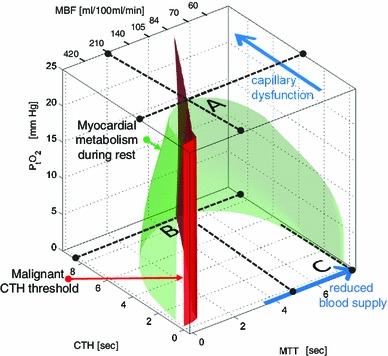
Metabolic thresholds. The green surface indicates combinations of MTT (MBF is shown on the upper *x*-axis), CTH, and P_t_O_2_ that provide sufficient oxygen to meet the metabolic rate of the myocardium during rest. The *red plane* marks the boundary, left of which vasodilation reduces tissue oxygen availability (malignant CTH). The *blue arrows* indicate the principal ways in which myocardial oxygen availability can be reduced in disease: by reductions in MBF (by CAD), increases in CTH (capillary dysfunction), and combinations thereof—see text. Abbreviations: *CTH* capillary transit time heterogeneity, *MBF* myocardial blood flow, *MTT* mean transit time, *P*_*t*_ *O*_*2*_ = tissue oxygen tension, *CAD* coronary artery disease

If CTH continues to increase, the largest MMRO_2_^max^ that can be achieved by the tissue circulation gradually falls toward the actual metabolic needs of the resting myocardium—see Fig. 4. The green, three-dimensional surface on Fig. 7 corresponds to the combinations of MTT, CTH, and P_t_O_2_, that give rise to a tissue oxygen availability equal to the metabolic rate of oxygen of the myocardium during rest, MMRO_2_ = 10 mL O_2_/100 mL/min [1]. The surface is formed by joining the 10 mL/100 mL/min iso-contours (cf. Figure 5B) for P_t_O_2_ values between 0 and 25 mmHg. The resulting green half-cone therefore contains hemodynamic conditions that can support metabolic needs of the myocardium during rest. The red plane marks the boundary to malignant CTH.

Assuming that P_t_O_2_ is kept constant at 25 mmHg, the point labeled A indicates the critical level of capillary dysfunction for this P_t_O_2_ in myocardium: if CTH increased beyond this value, oxygen availability would no longer be able to meet the metabolic needs of resting myocardium. As described above, the tissue oxygen tension will gradually fall as oxygen availability approaches the metabolic needs of the tissue, and the figure illustrates how the more efficient extraction permits the maintenance of oxygen availability for a wider range of MBF and CTH values: the green half-cone becomes wider towards its base, indicating that at relative tissue hypoxia, myocardial oxygenation can be maintained across a wider range of CTH and (lower) MBF values.

If CTH increases further, it reaches a critical limit (broken line parallel to the MTT axis at P_t_O_2_ = 0), at which tissue oxygen tension is negligible and MTT maximizes MMRO_2_^max^ (Labeled B). Note that, at this point, the metabolic needs of tissue cannot be supported unless MTT is prolonged to a threshold of approximately 5 s, corresponding to MBF = 84 mL/100 mL/min. In other words, the gradual reduction in tissue oxygen availability owing to progressive capillary dysfunction (increase in CTH) can be compensated for by gradual reductions in tissue oxygen tension and in resting MBF.

Figure 7 also allows us to analyze myocardial oxygenation after a sudden reduction in MBF, for example as a result of the partial obstruction of an upstream epicardial artery. Myocardial oxygen availability remains above the metabolic demands of resting myocardium until MTT exceeds 8 s, corresponding to an MBF threshold of 53 mL/100 mL/min, provided that CTH is negligible or moderate (Label C).

### Triggers of myocardial infarction and angina in conditions of high CTH

The model of myocardial oxygen availability presented here predicts that not only reductions in MBF, but also increases in CTH, can trigger a critical lack of oxygen and thereby angina and/or acute cardiac syndrome. Furthermore, increasing capillary dysfunction is predicted to reduce tissue oxygen reserves towards that of the myocardium during rest, in a gradual fashion. Theoretically, minor reductions in MBF or slight increases in CTH are therefore sufficient to trigger reductions in tissue oxygen tension, and thereby angina and systolic dysfunction. In principle, angina is therefore predicted to be either ‘arterial’ in origin (triggered by reductions in MBF, for example due to an atherosclerotic stenosis or small vessel disease), or ‘capillary’, triggered primarily by elevated CTH. In the latter case, MBF is predicted to be moderately reduced, having adapted itself to maximize myocardial oxygenation, cf. Figs. 4 and 7. Therefore, tissue affected by either a chronic or a sudden increase in CTH is predicted to appear hypoperfused when compared to tissue with unaffected by CTH, even in the absence of any vessel stenosis.

The extent to which episodes of elevated CTH can trigger angina or systolic dysfunction remains unclear. Dehydration, and infections accompanied by elevated viscosity due to an increased neutrophil count and endothelial adhesion [69], would be expected to elevate CTH and thereby cause critical reduction in myocardial oxygen availability. The seasonal variation in cardiovascular deaths [96] has indeed been linked to increased neutrophil count in relation to winter respiratory infections [116]. This mechanism might contribute to the hypothesized relation between acute infections and cardiovascular deaths, and between chronic infections and the development IHD [67].

### Tissue injury during myocardial ischemia: the predicted effect of capillary flow disturbances

The extended flow-diffusion equation predicts that tissue oxygen availability in hypoperfused tissue is affected by the extent of the MBF reduction, as well as CTH. While the level of hypoperfusion would be expected to remain constant until successful recanalization therapy, changes in capillary flow patterns during the ischemic period could cause tissue oxygen availability to deteriorate. The extent of tissue damage during the ischemic period may therefore depend on factors other than the vascular occlusion, and potentially, be modified prior to hospitalization. Capillary flows are indeed known to undergo profound changes during experimental ischemia owing to capillary plugging, endothelial damage, capillary leakage, pericapillary edema, and reductions in local flow [32].

### Reperfusion injury: the putative roles of capillary occlusions and capillary compression/constriction

Successful normalization of MBF after episodes of myocardial ischemia is often accompanied by reperfusion injury (RI) which accounts for up to 50 % of myocardial damage in animal models, and may account for a proportion of the deaths and cardiac failures that occur in humans, in spite of optimal recanalization therapy [119].

The extended flow-diffusion equation predicts that in order for tissue reperfusion to restore myocardial oxygen availability to its pre-ischemic level, both MBF and CTH must be restored to their pre-ischemic values. This prediction re-emphasizes the notion that recanalization must be accompanied by the reversal of any capillary plugging, pericyte constriction, endothelial swelling, and edema-driven capillary compression that may have evolved during the ischemic/hypoxic period, as well as any capillary flow disturbances that may have resulted from the lysis or mechanical removal of upstream clots [45]. If not, any residual capillary obstructions, or elevated CTH across perfused capillaries, would be predicted to prevent optimal re-oxygenation, and possibly to attenuate MBF or MFR to optimize myocardial oxygen availability, cf. Figs. 4 and 7. Both endothelial edema and granulocyte entrapments persist after myocardial reperfusion [119] and would hence be expected to cause elevated CTH levels and result in incomplete re-oxygenation. The passage of plasma through the microcirculation is predicted to be less disturbed by capillary changes than that of erythrocytes, due to their size and capillary adhesion properties. The prediction that capillary flow disturbances contribute to reperfusion injury is therefore consistent with the finding that the use of ‘free’ hemoglobin as an oxygen carrying perfusate reduces reperfusion injury [16].

The malignant CTH phenomenon also implies that, if CTH is not immediately restored upon reperfusion, OEF^max^ will remain low for short MTT, and the sudden restoration of flow through fully dilated arteries and arterioles could therefore, paradoxically, cause immediate, severe tissue hypoxia and tissue damage. On the other hand, the increase in perfusion pressure after recanalization would be expected to augment the dilation of capillaries and help restore homogenous capillary flows. Reperfusion injury is greatly reduced in animal models if perfusion and perfusion pressure is gradually increased to pre-ischemic values over a few minutes, as opposed to uncontrolled, hyperemic reperfusion [76, 81]. Similarly, post-conditioning (episodes of interrupted flow after recanalization) seemingly improves tissue salvage, electrical function and outcome in patients [97]. The extent to which this beneficial effect is related to the restoration of capillary patency and CTH remains unclear.

## Discussion

The extended flow-diffusion equation may help us widen our understanding of IHD and MVD from being conditions characterized by limited MBF, to also take the effects of elevated CTH on myocardial oxygen availability into account. In the resulting understanding of the etiopathogenesis of IHD and MVD, angina and acute coronary syndrome are preceded not only by atherosclerosis and/or small vessel disease, but also by changes in capillary morphology or blood viscosity that cause CTH to increase. The resulting changes in oxygen extraction efficacy are predicted to require adaptations of both resting MBF and MBF responses during exercise to meet the metabolic needs of myocardial oxygen metabolism. In fact, a gradual increase in CTH is predicted to require adaptations that are consistent with initial findings of elevated resting MBF and of gradual reductions in CFR in MVD risk factors. The extended flow-diffusion equation predicts that heart failure is the result of reductions in MMRO_2_^max^ to levels below the metabolic needs of the myocardium, either as a result of result of reductions in MBF (the traditional understanding of IHD), as a result of elevated CTH levels, or both. Importantly, the equation predicts that both the restoration of MBF, and of capillary flow patterns, represent key aspects of reducing ischemic damage and reperfusion injury after myocardial ischemia. Therapeutic means of maintaining capillary perfusion, both prior to hospitalization and in relation to recanalization, could therefore prove to be important in the management of acute cardiac syndrome.

### Can capillary dysfunction elicit endothelial dysfunction and attenuate MBF responses?

The endothelial dysfunction and reduced CFR observed in patients with cardiovascular risk factors, IHD, and MVD, are predicted by our model to represent necessary adaptations to increasing CTH as hyperemia gradually fails to increase myocardial oxygen availability. The ways by which capillary dysfunction can attenuate upstream vasodilation (endothelial dysfunction), however, remain unclear. The coordination of microvascular function is controlled in large parts by efficient, bidirectional signaling among endothelial cells, who act as metabolic ‘sensors’ [27, 50, 94, 95]. The extent to which connexins are involved in endothelial dysfunction in relation to tissue hypoxia has only recently been studied [87]. A defining feature of capillary dysfunction is that tissue hypoxia gradually develops as vasodilation can no longer support the metabolic needs of the tissue during exercise. Such reductions in tissue oxygen tension would be expected to lead to the activation of hypoxia-inducible transcription factors (HIF), which in turn initiates a number of adaptations in tissue to better tolerate low oxygen levels. One of these is the up-regulation of nicotinamide adenine dinucleotide phosphate (NADPH) oxidase (NOX) levels [123]. NOX is a major source of superoxide and free radicals in the vasculature [9]. Endothelial dysfunction is indeed mediated by elevated levels of superoxide anions in the vessel wall, and parallel depletion of nitric oxide (NO). As a consequence, capillary dysfunction could, in principle, elicit upstream endothelial dysfunction via a hypoxia-sensitive mechanism. Of note, the up-regulation of hypoxia-inducible factor type 1 HIF-1 also leads to the initiation of inflammatory processes via activation of nuclear factor κB (NF-κB) [30]. Therefore, tissue inflammation may be an additional, obligatory companion to capillary dysfunction—see Fig. 4.

Long-term ROS exposure of artery and arteriole walls to ROS and low NO are known to cause remodeling and thickening of the vessel walls [104]. With more prolonged oxidative damage, vascular smooth muscle cells may degenerate and develop abnormal focal constrictions that result in additional narrowing of their luminal diameters. These adaptations tend to attenuate flow and flow responses, and may therefore, paradoxically, protect tissue from hypoxic episodes caused by increases in blood flow. We have speculated that vascular ROS production may have another, more severe side-effect: ROS and peroxynitrate are likely to reach capillaries immediately downstream, where peroxynitrate can cause further damage to the capillary wall, while both increased ROS levels [120] and reduced NO levels [37, 38] are believed to cause pericyte constriction. As a consequence, endothelial dysfunction may result in abnormal constriction of additional capillaries, further elevating CTH, and thereby further exacerbate the detrimental lack of oxygen in a vicious cycle.

### Implications for the prevention and management of IHD

Elevated superoxide levels in vessels and tissue are likely to deplete capillary NO levels in the myocardium, while tissue hypoxia causes a lack of substrate for NO production via tissue NO-synthases. Means of restoring capillary NO levels or preventing the constriction of capillary pericytes may therefore prove cardioprotective in IHD, and during acute cardiac syndrome. This is consistent with findings that nitroglycerine appears to reduce tissue hypoxia by altering microvascular flow patterns, but without changing overall vascular resistance [53]. Tissue nitrite stores can be reduced to NO during ischemia or hypoxia without the need of tissue oxygen as a substrate. The administration of nitrite during cardiac ischemia has indeed been shown to be cardioprotective in animal studies, and circulating levels of nitrite are thought to be related to cardiovascular risk [17]. Green leafy vegetable is a major dietary source of nitrite in humans [66], and the intake of these has been shown to be inversely related to the incidence of myocardial infarctions [49].

### Biophysical modeling of myocardial oxygenation and oxygen consumption

Oxygen transport models have been used extensively in the attempt to understand the relation between MBF and metabolism, and to derive myocardial oxygen metabolism based on dynamic recordings of [^15^O] in tissue or in venous blood [24, 65, 115] see Fig. 3B. Myocardial perfusion is very heterogeneous when measured at a millimeter scale, a phenomenon termed micro-heterogeneity to distinguish it from regional and transmural flow differences [3, 5–7, 36, 57, 92, 107]. To account for flow heterogeneity within the tissue volume being modeled, oxygen transport models therefore typically include flow distribution and vascular dispersion terms which can then be calibrated by measured relative dispersion (RD) values from tissue samples [58]—see Fig. 3B. The figure also shows the capillary-tissue oxygen exchange and oxygen utilization model developed by Li et al. [65], in which the binding of oxygen to hemoglobin and myoglobin is accounted for. This model has been utilized to fit dynamic ^15^O_2_ data in isolated hearts, and the predicted myocardial oxygen utilization shown to be in good agreement with direct measurements using Fick’s principle [92]. We have previously used the model in Fig. 3B (left panel) to model capillary flow heterogeneity, assigning different numerical weights and fractional flows to the parallel paths in Fig. 3B [78]. With its large number of parameters, however, this exhaustive model does not lend itself to an intuitive analysis of how MBF, CTH, and tissue oxygen levels, respectively, permit a given myocardial oxygen utilization.

The extended flow-diffusion equation models oxygen availability with as few parameters as possible in order to examine how blood flow, CTH and tissue oxygen tension, combined, can support a given metabolic demand, while permitting intuitive visualization of these limitations in three-dimensional plots such as Fig. 7. Similar to the approach of Li et al., who used a lagged normal density function to describe flow heterogeneity, we used a gamma variate function to describe transit time heterogeneity, in order to parameterize CTH by a single parameter—in this case the standard deviation of transit times. Similarly, we incorporated the binding of oxygen to hemoglobin in blood. Myocardial capillaries are organized in a complex, interconnected three-dimensional network [8, 54]. While spatially distributed models such as that of Li et al. requires assumptions on interstitial oxygen diffusion within this complex topology, and the degree of oxygen binding to myoglobin, we assumed a uniform interstitial oxygen tension. By having tissue oxygen tension as our model’s third variable (see Fig. 7), the model clarifies the physiological distinction between myocardial ischemia (low blood flow) and myocardial hypoxia, which is directly related to IHD symptoms. As shown in Fig. 3, neither model directly models the interconnectedness of myocardial capillaries, a feature better captured by network models [115]. Kiyooka et al. imaged epicardial capillary flow patterns in dogs and found a significant increase in the diameter of, and blood flow through, cross-connecting capillaries during reactive hyperemia [59]. The flow through dilated cross-connecting capillaries would be expected to reduce flow differences among parallel capillary paths, and hence to facilitate the homogenization of capillary flow patterns during reactive hyperemia. According to our model and the observations by Rose et al. [89], this topological feature of the myocardial capillary bed thus seems to facilitate the maintenance of constant oxygen extraction fraction across a wide range of flow values in the heart. More complex features of the myocardial microcirculation, such as its compression during systole, may also be of relevance to future biophysical models of myocardial oxygen transport [35].

### Capillary recruitment

The classical flow-diffusion equation [88], as well as more complex models such as that of Li et al. [65], predict that the extraction of diffusible substances is limited by blood flow and the capillary permeability (P) multiplied by their surface area (S). Assuming that capillary permeability is constant for a given molecule, these models therefore imply that the net extraction of a given substance can be increased either by increasing blood flow, or by capillary recruitment (opening of previously closed capillaries to increase capillary surface area) [62]. Applying the oxygen transport model of Li et al. to dynamic ^15^O_2_ data, Schwanke et al. [92] found that satisfactory data fits could only be achieved by assuming that PS for oxygen increases linearly with MBF during a threefold increase in flow, and a sixfold increase in oxygen utilization. Although a slight increase in capillary blood volume is observed during hyperemia, such an increase in the number of perfused capillaries is contradicted by observations [59]. The extended flow-diffusion equation offers a crucial advantage over existing models by offering an explanation to this ‘recruitment paradox’: the physiological effect of CTH is to modify the extent to which diffusible tracers can exchange with tissue through the capillary wall, and thereby their ‘apparent’ capillary surface area, even for a fixed, ‘physical’ PS. We showed that the ‘apparent’ PS one would observe in the heart is given by PS = −MBF·ln(1−OEF^max^) [52]. Provided that myocardial metabolism and hemodynamics are coupled, so that OEF = OEF^max^, our model therefore predicts that apparent PS for oxygen in the myocardium (where OEF is remarkably constant) increases linearly with MBF, as Schwanke et al. [92] observed and had to assume in order to fit experimental data to their model.

### Capillary transit time heterogeneity versus myocardial flow heterogeneity

The CTH phenomenon, and the potential ‘oxygen loss’ due to functional shunting through capillary paths with short transit times, has received much less attention [47, 89] than myocardial flow heterogeneity on the millimeter scale and beyond [3, 5–7, 36, 57, 92, 121] due to the initial suspicion that areas of low perfusion were prone to ischemic damage. Myocardial micro-heterogeneity is typically reported as the standard deviation of flow values within a certain tissue volume, and remains relatively constant (30 % of the resting flow) across tissue volume sizes and species [6]. The heterogeneity in flow appears to be linked to the topology of the vascular tree [48], and detailed studies of the oxygen supply–demand balance within the myocardium suggest that metabolic needs are in fact met [124] in spite of the low perfusion values. The relation between flow heterogeneity and the regulation of arterial and arteriolar tone is reviewed in greater detail in Ref. [101]. We speculate that CTH, and thereby OEF^max^, is actively regulated, possibly via pericyte dilation [39], to meet the metabolic needs of the tissue for a given MBF and tissue oxygen tension. This notion is consistent with the observation that flow heterogeneity is paralleled by considerable heterogeneity in the oxygen saturation of small myocardial veins [114], with high OEF at low flow rates [92]. Similarly, metabolic needs in the right ventricle are seemingly met to some extent by increased OEF rather than MBF [40].

### Measurement of CTH, MTT, and capillary recruitment in the myocardium

We hypothesize that the model parameters of the extended flow-diffusion model (MTT, CTH, and tissue oxygen tension) hold information about myocardial oxygen availability that cannot be gleaned from MBF measurements alone. The coronary microcirculation can be assessed by a number of techniques, albeit direct observation of capillary flow patterns in the myocardium itself remains challenging [59, 83]. Myocardial blood transit time characteristics can in principle be determined by routine cardiac MRI, CT, or ultrasound ‘bolus tracking’ perfusion measurements using contrast agents with little or no first-pass extraction [63, 113]. We have shown that this approach permits reliable retrieval of MTT and CTH as parametric maps at the typical signal-to-noise ratio of clinical perfusion MRI in the brain [75]. Transit time distributions retrieved by this approach are characteristics of individual voxels, which for state-of-the-art cardiac perfusion MRI are 1.7 × 1.9 × 10 mm [73]. Given the heterogeneity of myocardial flows over short distance scales, the stability of such measurements should be carefully evaluated to ensure that they reflect CTH of uniformly perfused tissue. Qualitatively, increased CTH would be expected to result in delayed tissue-clearance of contrast medium during angiographic procedures. This phenomenon is indeed observed in some patients suspected of MVD [10].

### Perspectives

The presence of age- or risk-factor-related changes in capillary morphology constitutes a key difference between human IHD and the animal models that are typically used in the study of myocardial ischemia and reperfusion injury. Given the putative effects of capillary flow disturbances described here, this difference could affect the extent to which cardioprotective strategies developed in animal models with normal regulation of CTH translate into successful human therapies. We speculate that the use of animal models with capillary changes characteristic of human disease (spontaneously hypertensive rats, diabetic animals, pharmacological degradation of the glycocalyx, and so forth) may provide more realistic models of human disease.

This review re-emphasizes the importance of biophysical modeling as a means of understanding the relation between myocardial blood supply and the metabolic needs of the myocardium. Combining such models with cutting-edge imaging techniques, we speculate that studies of the importance of capillary flow distributions in MVD, myocardial ischemia, and reperfusion injury may be within reach in both animal models and humans. Meanwhile, we must develop a thorough understanding of capillary function, including that of the glycocalyx, of endothelial cells, and of the regulation and pharmacological modulation of pericyte tone.
